# Novel homozygous mutations in *TXNDC15* causing Meckel syndrome

**DOI:** 10.1002/mgg3.2343

**Published:** 2023-12-29

**Authors:** Tianqin Deng, Yuli Xie

**Affiliations:** ^1^ Reproductive Medical Center Shenzhen Maternity & Child Healthcare Hospital Shenzhen People's Republic of China; ^2^ Neonatal Screening Center Shenzhen Maternity & Child Healthcare Hospital Shenzhen People's Republic of China

**Keywords:** ciliopathy, Meckel syndrome, preimplantation genetic testing, *TXNDC15* gene

## Abstract

**Background:**

Meckel syndrome (MKS) is the most severe form of an autosomal recessive ciliopathy and is clinically characterized by occipital encephalocele, severely polycystic kidneys, and postaxial polydactyly (toes). The association of TXNDC15‐related MKS has been reported. We report the case of a homozygous mutation in the TXNDC15 gene, causing MKS14 in the Chinese population.

**Methods:**

The fetal skin tissue and parental peripheral blood were retained for whole‐exome sequencing and Sanger sequencing, which investigated the potential pathogenic variants associated with MKS.

**Results:**

The fetus was homozygous for a mutation in the TXNDC15 gene (NM_024715.3), specifically c.560delA (p.Asn187llefsTer4), and both parents were heterozygous for this mutation.

**Conclusion:**

Our study identified a new mutation that adds to the mutational landscape of MKS, which provide a basis for genetic counseling and the selection of reproductive options.

## Graphical abstract

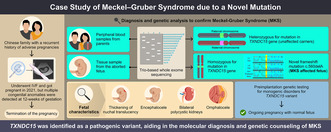

## 1 INTRODUCTION

Meckel syndrome (MKS, OMIM#249000) is the most severe form of an autosomal recessive ciliopathy and is clinically characterized by occipital encephalocele, severely polycystic kidneys, and postaxial polydactyly (toe). Typically, fetuses with MKS can be diagnosed through ultrasound in the first and second trimesters (Hartill et al., 2017). The prognosis for MKS is poor, with intrauterine death being common and the fetus dying either in utero or within hours to days after birth, and there is no cure. In this study, whole‐exome sequencing of probands and their parents was performed to identify the genetic cause of MKS and provide a basis for genetic counseling and the selection of reproductive options.

## 2 MATERIALS AND METHODS

### 2.1 Objective

**FIGURE 1:**
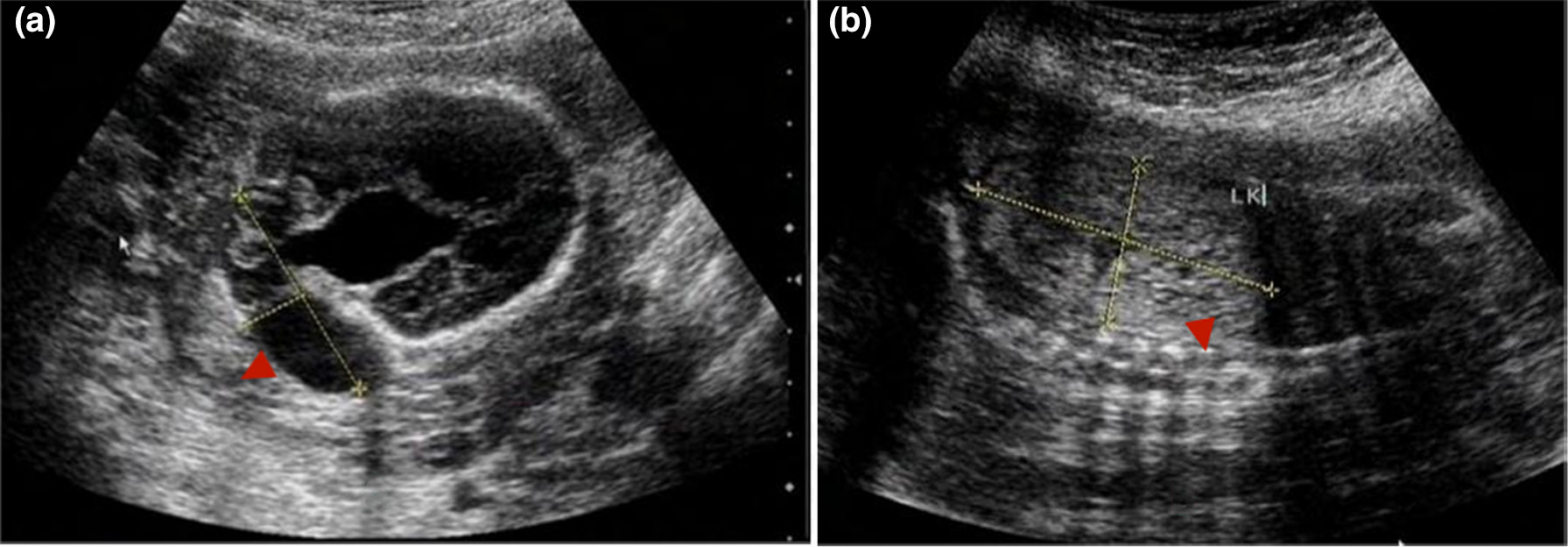
(a) Ultrasound image of fetal meningoencephalocele. The meningoencephalocele was approximately 4.4 cm × 1.3 cm in size (red arrowhead indicates meningoencephalocele). (b) Ultrasound image of the left kidney of the fetus. The left kidney was approximately 5.1 cm × 2.7 cm in size (red arrowhead indicates left kidney).

The patient was a 36‐year‐old female in a nonconsanguineous marriage, gravida 4 para 0 abortus 4 (G_4_P_0_A_4_). In 2014, the patient miscarried at 5 weeks, and no embryonic karyotyping was performed. In 2015, an ultrasound examination of a fetus at 22 weeks revealed indications of fetal meningoencephalocele, bilateral polydactyly of the foot, and polycystic kidneys (Figure 1), suggesting MKS. After prenatal diagnosis, the pregnancy was terminated without fetal karyotyping. In 2017, the patient miscarried at 7 weeks, and no embryonic karyotyping was performed. In 2021, ultrasound examination of a fetus at 12 weeks revealed developmental abnormalities, including a nuchal translucency thickness (NT) of 4.0 mm, an encephalocele 5 mm × 5 mm in size, an omphalocele 7 mm × 4 mm in size, polycystic kidneys (left kidney, approximately 11 mm × 7 mm in size; right kidney, approximately 12 mm × 6 mm in size; and multiple cystic cavities of different sizes observed in both kidneys), suggesting thickening of the NT and possible MKS. After genetic counseling, the patient elected to terminate the pregnancy. After labor induction, fetal skin tissue and parental peripheral blood were retained for whole‐exome sequencing to confirm the diagnosis. The study was approved by the Ethics Committee of the Shenzhen Maternal and Child HealthCare Hospital (No: SFYLS[2021]038), and all study participants provided informed consent in writing.

### 2.2 Methods

#### 2.2.1 Genomic DNA extraction

DNA was extracted from fetal tissue and parental peripheral blood using kits, as per the manufacturer's instructions.

#### 2.2.2 Whole‐exome sequencing and analysis

Genomic DNA was extracted using the GenCap Deafness capture kit (MyGenostics GenCap Enrichment Technologies). Whole‐exome library construction was performed with at least 3 μg of patient DNA using the Agilent SureSelect Human All Exon (Agilent Technologies (China) Co., Ltd.) kit. Capture probes were designed for exons, as well as 5′ and 3′ UTR regions, and hybridized to DNA library fragments of 150–200 bp. Next, human whole‐exon‐sequencing libraries were indirectly obtained based on binding between biotin on the probes and streptavidin‐coated magnetic beads, which was followed by enrichment of the magnetic beads. After constructing the library, an Illumina sequencer was used for sequencing. Finally, valid data were analyzed through bioinformatics analysis and the splitting and filtering of data. Exome variation data were generated by comparing the sequencing data with the hg19 human genome reference sequence.

#### 2.2.3 PCR amplification and Sanger sequencing

According to the reference sequence of *TXNDC15* gene (NC_000005.9), the upstream and downstream primer was designed at both ends of the pathogenic loci, which were analyzed via PCR amplification, PCR product purification, cycle sequencing, post‐sequencing purification, and capillary electrophoresis using the Applied Biosystems instrument, and finally, alignment of pathogenic loci was performed using the Minor Variant Finder software (Applied Biosystems).

## 3 RESULTS

**FIGURE 2:**
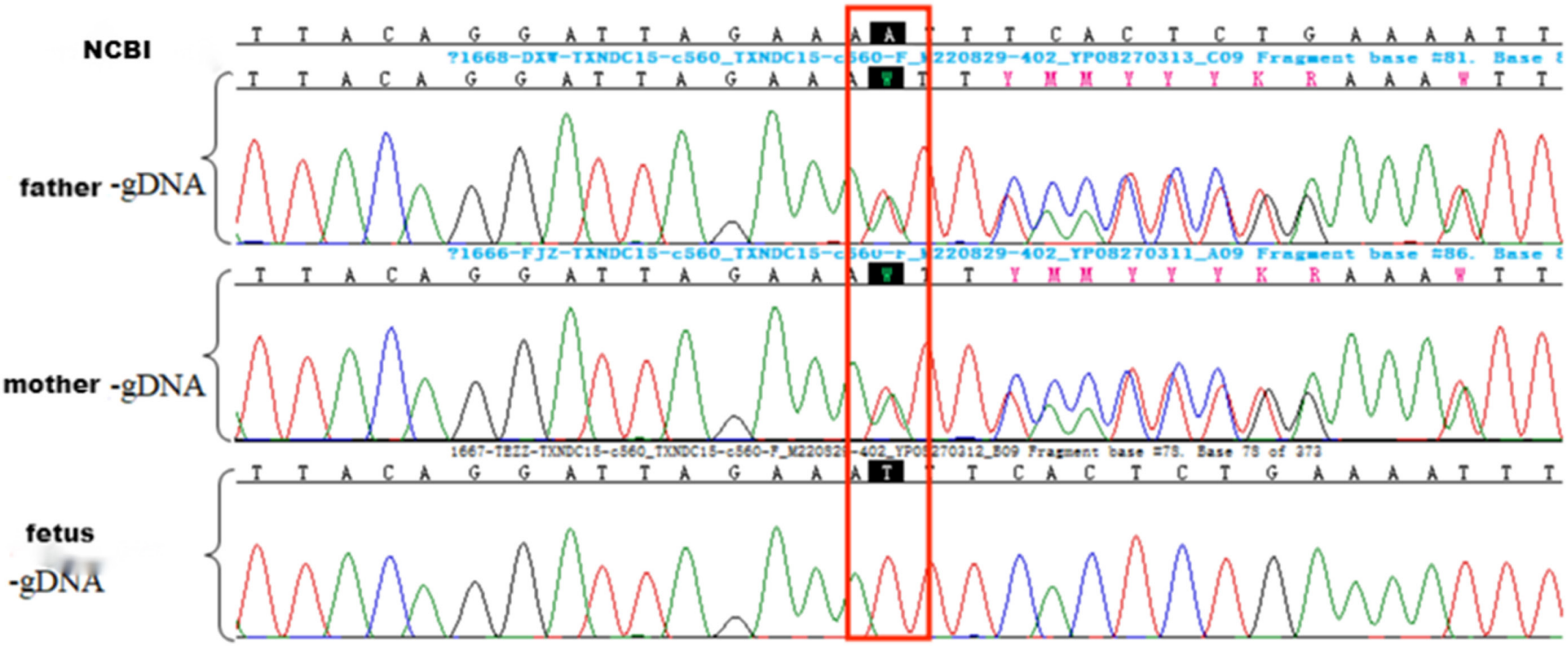
Sanger sequencing results of the *TXNDC15* gene in the family. Both the father and mother were heterozygous for the c.560delA mutation in the *TXNDC15* gene, and the fetus was homozygous for the c.560delA mutation in the *TXNDC15* gene. Red boxes represent mutation loci. The first line is the TXNDC15 gene reference sequence(NC_000005.9).

Whole‐exome sequencing and Sanger sequencing revealed that the fetus was homozygous for a mutation in the *TXNDC15* gene (NM_024715.3), specifically c.560delA (p.Asn187llefsTer4), and both parents were heterozygous for this mutation (Figure 2). The *TXNDC15* gene is a causative gene of MKS, and c.560delA (p.Asn187llefsTer4) is a previously unreported novel frameshift mutation producing a nonfunctional truncated protein that affects protein function. It was not found in human exome databases, the 1000 Genomes Project reference panel, or the Human Gene Mutation Database. According to the standards and guidelines for the interpretation of sequence variants by the American College of Medical Genetics and Genomics (Richards S et al., 2015), the locus was classified as a pathogenic variant (PVS1 + PM2 + PP4). Ultrasound examination of the patient's second and fourth pregnancies indicated multiple fetal malformations (encephalocele, omphalocele, bilateral polycystic kidneys, and bilateral polydactyly of the foot) with a clinical phenotype consistent with that of MKS. In conjunction with the clinical phenotype of the fetus, the homozygous c.560delA (p.Asn187llefsTer4) mutation in the *TXNDC15* gene was considered the pathogenic variant in the fetus. After genetic counseling at our hospital, the couple selected preimplantation genetic testing for a monogenic disorder (PGT‐M) to block inheritance of the pathogenic gene and prevent pregnancy with an affected child.

## 4 DISCUSSION

In conclusion, the present study elucidated the genetic etiology of an MKS pedigree through whole‐exome sequencing, thus providing a basis for genetic counseling and the selection of reproductive options; it also identified a new mutation that adds to the mutational landscape of MKS. MKS, a severe autosomal recessive disorder, is one of the most severe forms of ciliopathy with typical clinical manifestations, such as meningoencephalocele, polycystic kidneys, polydactyly (toe), and hepatic bile duct fibrosis. MKS exhibits significant clinical heterogeneity, with abnormalities in the central nervous systems, cystic kidneys, and dilated, sclerotic bile ducts in the liver as the major clinical phenotypes (Logan et al., 2011). This family had two conceptuses with MKS, both presenting with meningoencephalocele and polycystic kidneys, but no manifestations of hepatic bile duct fibrosis, thus exhibiting clinical heterogeneity. The incidence of MKS is inconsistent according to reports; based on statistics, the worldwide incidence of MKS is 1/135,000 (Auber et al., 2007), and this is higher in families or countries with many consanguineous marriages, such as among Kuwaiti Bedouins (1/3530) (Teebi et al., 1992) and Saudi Arabs (1/3500) (Teebi & Teebi, 2005).

MKS‐causative genes have been identified as follows: *MKS1*, *TMEM216*, *TMEM67*, *CEP290*, *RPGRIP1L*, *CC2D2A*, *NPHP3*, *TCTN2*, *B9D1*, *B9D2*, *TMEM231*, *TMEM107*, *TXNDC15*, *C5orf42*, and *CSPP1d*. *KIF14* and *CEP55* are strong candidates for causal genes. At present, the molecular cause of only 60% of cases can be explained by mutations in the known MKS‐associated genes (Moreno‐Leon et al., 2022). MKS14 (OMIM 619879) is caused by homozygous or compound heterozygous mutations in the *TXNDC15* gene (617778) on chromosome 5q31.1. This confirms that biallelic pathogenic variants of *TXNDC15* cause MKS in human fetuses (Radhakrishnan et al., 2019). In 2016, Shaheen et al. (2016) reported three cases of MKS14, all caused by homozygous mutations in *TXNDC15*, with mutation loci including *TXNDC15* (NM_024715.3c.672_686del) (Ser225_His229del), *TXNDC15* (c. 103 + 1G‐A, NM_024715.3), and *TXNDC15* (NM_024715.3c.956dupT) (Ser321LysfsTer15). In 2019, Ridnõi et al. (2019) reported a case of MKS14 caused by compound heterozygous mutations in the *TXNDC15* gene, acquired from each parent, with mutations at the loci *TXNDC15* (NM_024715.3 c.211dup) and (NM_024715.3 c.635 T‐C). Both variants were found to be present at very low minor allele frequencies (0.029% and 0.00041%, respectively) in the gnomAD database. In 2019, Radhakrishnan et al. (2019) reported a case of MKS14 in an Indian fetus caused by a homozygous mutation in the *TXNDC15* gene at the locus *TXNDC15* (NM_024715.3c.844C‐T). The unaffected parents were heterozygous for the mutation, which was determined to be present at a very low minor allele frequency (0.000008128) in the gnomAD database.

Here, we report a case of MKS diagnosed through fetal ultrasound diagnosis in which whole‐exome sequencing of fetal tissue and parental peripheral blood revealed the homozygous NM‐024715.3c.560delA (p. Asn187llefsTer4) mutation in the *TXNDC15* gene in the fetus and the same heterozygous mutation in both parents. This was determined to be a frameshift mutation in the *TXNDC15* gene that was inherited from both phenotypically normal parents. According to the ACMG guidelines, the mutation was classified as pathogenic, with c.560delA being a previously unreported novel mutation. After confirming the pathogenicity of the genetic variant, the couple was offered genetic counseling, which provided strong evidence for the selection of reproductive options and the subsequent prenatal diagnoses, and in the end, the couple selected PGT‐M to block inheritance of the pathogenic gene. PGT‐M has been previously used to block inheritance of pathogenic genes causing MKS1 (Lin et al., 2022) and MKS5 (Zhang et al., 2022).

Currently, the fetal diagnosis of MKS is performed primarily through ultrasound screening and genetic diagnoses. In pregnant women without a family history of MKS, most cases are detected via ultrasound screening in the first and second trimesters. Transabdominal ultrasonography, performed at 10–14 weeks of gestation, has been shown to have the ability to successfully detect several fetal anomalies associated with MKS (Jones et al., 2014). For couples with a family history, MKS has an autosomal recessive inheritance pattern with a risk of recurrence as high as 25% (Srivastava et al., 2022). Such couples can choose to prepare for pregnancy naturally with the help of initial screening via ultrasound in early pregnancy, and if no abnormality is observed based on the first‐trimester ultrasound, amniocentesis is performed in the second trimester to further determine whether the fetus is carrying the causative gene. Alternatively, the couple can elect to use PGT‐M for the selection of embryos for transfer that do not carry the causative gene, to block inheritance of the causative gene (Lee et al., 2020). In conclusion, the present study elucidated the genetic etiology of an MKS pedigree through whole‐exome sequencing, providing a basis for genetic counseling and the selection of reproductive options, in addition to identifying a new mutation that adds to the mutational landscape of MKS.

## AUTHOR CONTRIBUTIONS

Tianqin‐Deng collected and analyzed the cohort data and wrote the initial draft of the manuscript. Yuli‐Xie provided and analyzed clinical data of the patients, and reviewed the manuscript.

## FUNDING INFORMATION

This work was supported by the Shenzhen Science and Technology Innovation Program (No: JCYJ20220530155011024).

## CONFLICT OF INTEREST STATEMENT

The authors declare that they have no conflict of interest.

## ETHICS STATEMENT

The study was approved by the Ethics Committee of the Shenzhen Maternal and Child HealthCare Hospital (No: SFYLS[2021]038) and informed consent of the participating individuals or their legal guardians was obtained.

## Data Availability

All analyzed data consists of patient's personal data and stored by regulations of the institutions. On request is possible to share anonymised data.
